# *Cuspidothrix* Is the First Genetically Proved Anatoxin A Producer in Bulgarian Lakes and Reservoirs

**DOI:** 10.3390/toxins14110778

**Published:** 2022-11-10

**Authors:** Maya Stoyneva-Gärtner, Katerina Stefanova, Blagoy Uzunov, Mariana Radkova, Georg Gärtner

**Affiliations:** 1 Faculty of Biology, Department of Botany, Sofia University, BG-1164 Sofia, Bulgaria; 2 AgroBio Institute, Bulgarian Agricultural Academy, BG-1164 Sofia, Bulgaria; 3 Institute of Botany, Innsbruck University, A-6020 Innsbruck, Austria

**Keywords:** Cyanobacteria, Cyanoprokaryota, cyanotoxins, ecosystem health, human health, phytoplankton, very fast death factor

## Abstract

The paper presents the first proof of planktonic cyanoprokaryote genus *Cuspidothrix* as an anatoxin A (ATX) producer in Bulgarian wetlands. The results from polymerase chain reaction (PCR) obtained from two summer sampling campaigns in 26 selected lakes and reservoirs demonstrated presence of the *anaC* gene, responsible for ATX production in 21 strains of the genus. They were found in three waterbodies sampled in 2018 (coastal lake Vaya, coastal reservoir Poroy, inland reservoir Sinyata Reka) and in four waterbodies sampled in 2019 (inland reservoirs Duvanli, Koprinka, Plachidol 2, Sinyata Reka). The detected genetic diversity generally corresponds to the observations conducted by conventional light microscopy, by which we distinguished three species of *Cuspidothrix* (*Cuspidothrix issatschenkoi*, *Cuspidothrix elenkinii* and *Cuspidothrix tropicalis*, the latter considered alien in the country). Eleven strains showed high similarity to two sequences of *C. issatschenkoi* available from the National Centre for Biotechnology Information (NCBI). Ten other strains assembled in a group, which—in lack of available from NCBI genetic sequences—were presumed related to *C. tropicalis* and *C. elenkinii* after comparison with the results from light microscopy. *Cuspidothrix* strains found in Bulgarian waterbodies showed high genetic similarity to those isolated and sequenced from Asia (Japan, China) and Northern Europe (Norway, Finland).

## 1. Introduction

Anatoxin A (ATX) and its analogues, anatoxins (ATXs), are alkaloid neurotoxins produced by more than 40 species of oxygenic photosynthetic prokaryotes from the phylum Cyanoprokaryota (blue–green algae)/Cyanobacteria) [1,2,3]. These toxigenic species occur mostly in freshwater, forming algal mats on the bottom of the waterbodies, or are floating in the water column. They were primarily referred to the following filamentous genera: *Anabaena*, *Aphanizomenon*, *Blennothrix*, *Chrysosporum*, *Cuspidothrix*, *Cylindrospermum*, *Dolichospermum*, *Kamptonema*, *Lyngbya*, *Microcoleus*, *Microseira*, *Moorea*, *Nostoc*, *Oscillatoria*, *Planktothrix*, *Phormidium*, *Raphidiopsis* (Syn. *Cylindrospermopsis*), *Sphaerospermopsis* and *Tychonema* [1,2,3,4,5,6,7,8,9,10,11], but two coccal genera, colonial *Microcystis* and *Woronichinia*, were also proved to be ATX producers [1,12,13]. The ability of cyanoprokaryotes to produce ATX is generic- and species-dependent, but one and the same species may comprise strains that are atoxic, while others can produce toxic metabolites [1,3]. Moreover, the strain specificity may contain a quantity of toxic compounds that can vary up to four orders of magnitude in different strains, thus explaining why high toxin concentrations could be achieved in nature even with low abundance from their producers (for details see [2]). In addition, almost all conducted studies indicated that presence of genes, responsible for the biochemical synthesis of certain toxins, is not sufficient for real toxin production and release in natural waters because gene expression is influenced by a variety of factors (e.g., [1]). An additional complication comes from the fact that the same species and even the same strain may contain multiple toxin operons and can produce diverse toxins from one “target” group (e.g., the neurotoxins ATX and saxitoxin) or even combinations of toxins from different groups (e.g., hepatotoxins and neurotoxins), depending on the conditions [12,13,14,15]. Therefore, assuming a given genus, species, or strain to be atoxic or toxic without proper investigation seems to be quite hazardous [13].

Physiological and toxicological research showed that ATX mimics the neurotransmitter acetylcholine, easily binds to its receptors of the boundary synapses located between nerve and muscle tissue, and stimulates them to release signals for muscle contractions in a persistent way [2,16]. Thus, ATX provokes neurotoxicoses in dogs, cattle and some wild animals, expressed in cramping, seizures and asphyxia with subsequent acute death [1,3,4,16,17,18,19,20,21,22,23,24]. The lethality can take 2–4 min in mice, and ATX was eloquently named “very fast death factor,” commonly abbreviated as VFDF [25,26,27,28]. Despite the fact that the effects for a long time were primarily related with fatal animal poisonings, ATX and its analogues (mainly homoanatoxin), may seriously impact human health through exposure to drinking water, recreational activities and seafood [2,3,29,30,31]. Consideration of the accumulated knowledge on their potential lasting implications led to the inclusion of ATXs in WHO *Guidelines for Drinking-Water Quality* and *Guidelines for Safe Recreational Water Environments* [3]. There, the following provisional health-based reference values of “30 µg L^−1^ for acute or short-term exposure via drinking-water and 60 µg L^−1^ for recreational water exposure” for adults and of ca. “6 µg L^−1^ for short periods” for small children were mentioned [3] (p. 1).

Some studies have supposed that the importance of ATXs posed to human and environmental health is increasingly rising and highlighted the necessity of their consideration in risk assessment [32]. The detected worldwide spread of ATXs in surface waters has been enhanced by global warming and increasing eutrophication [10,22]. These factors are widely accepted as general driving forces for the production of diverse algal toxic compounds and for the troublesome ecological “success” of harmful algal blooms (for details see [2,13,33,34]). Despite the rising global importance of ATXs, there are regions and countries with little information on their occurrence and photosynthetic producers. One of these “white spots” on the global map is Bulgaria, a country on the Balkan Peninsula with about 9000 waterbodies, including important large drinking water reservoirs, small reservoirs used for irrigation, and recreation activities or as fisheries, but also different types of natural lakes, some of which are of global, regional, or national conservational importance [35].

During a 15-year period of investigations of toxins produced by Cyanoprokaryota (cyanotoxins) in Bulgaria, the occurrence of ATXs was reported only once [36]. They were found by application of HPLC in samples from the mountain reservoir Borovitsa, collected in July 2006 [37]. The present paper provides the first genetic evidence of the occurrence of potential ATX producers in the country. They were obtained after processing of phytoplankton samples collected during two sampling campaigns (summers of 2018 and 2019) from 26 selected according to their intensive use and importance for human health waterbodies in Central and Eastern Bulgaria. By polymerase chain reaction (PCR), 21 planktic strains related to the genus *Cuspidothrix*, which contained the specific ATX synthetase *anaC* gene, were identified in samples from the largest coastal lake, Vaya (2018), in the coastal reservoir Poroy (2018), in the large inland reservoir Koprinka (2019), as well as in the small inland reservoirs Duvanli (2019), Plachidol 2 (2019) and Sinyata Reka (2018, 2019). The comparison of morphological data, obtained by conventional light microscopy (LM) with the genetic sequences allowed us to extend the information on the distribution of species previously recorded in the country: *Cuspidothrix issatschenkoi* (Usachev) P. Rajaniemi, Komárek, R. Willame, P. Hrouzek, K. Kastovská, L. Hoffmann et K. Sivonen and *C. elenkinii* (Kisselev) P. Rajaniemi, J. Komárek, R. Willame, P. Hrouzek, K. Kastovská, L. Hoffmann et K. Sivonen. In addition, we provide data on the spread of the new (for Bulgaria) tropical species, *C. tropicalis* (Horecká et Komárek) Rajaniemi P., J. Komárek, L. Hoffmann, P. Hrouzek, K. Kastovská et K. Sivonen, and hypothesize its alien character.

## 2. Results

### 2.1. Results from Light Microscopy Observations

Conventional LM investigations of the phytoplankton samples collected in 2018 revealed the presence of 85 species of Cyanoprokaryota in all investigated waterbodies. Out of them, 58 cyanoprokaryotes were identified in the three waterbodies, in which strains with *anaC* genes were detected by PCR: 53 in Vaya, 8 in Poroy and 7 in Sinyata Reka. Among all determined cyanoprokaryotes, 39 species were from the following 10 genera, known as ATX producers: *Anabaena*, *Aphanizomenon*, *Chrysosporum*, *Cuspidothrix*, *Dolichospermum*, *Microcystis*, *Oscillatoria*, *Phormidium*, *Planktothrix* and *Raphidiopsis*. In accordance with the obtained PCR data, we report only results concerning findings on *Cuspidothrix*.

**Figure 1:**
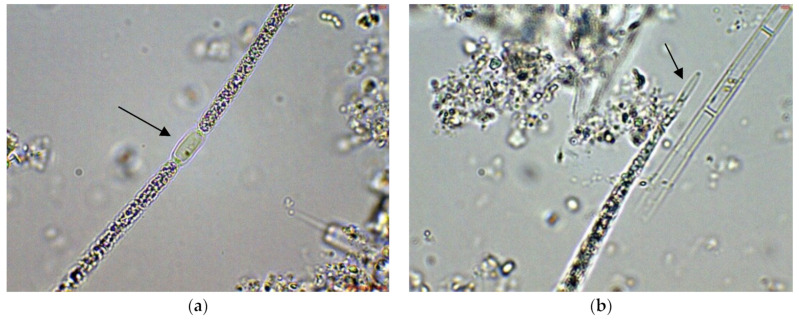

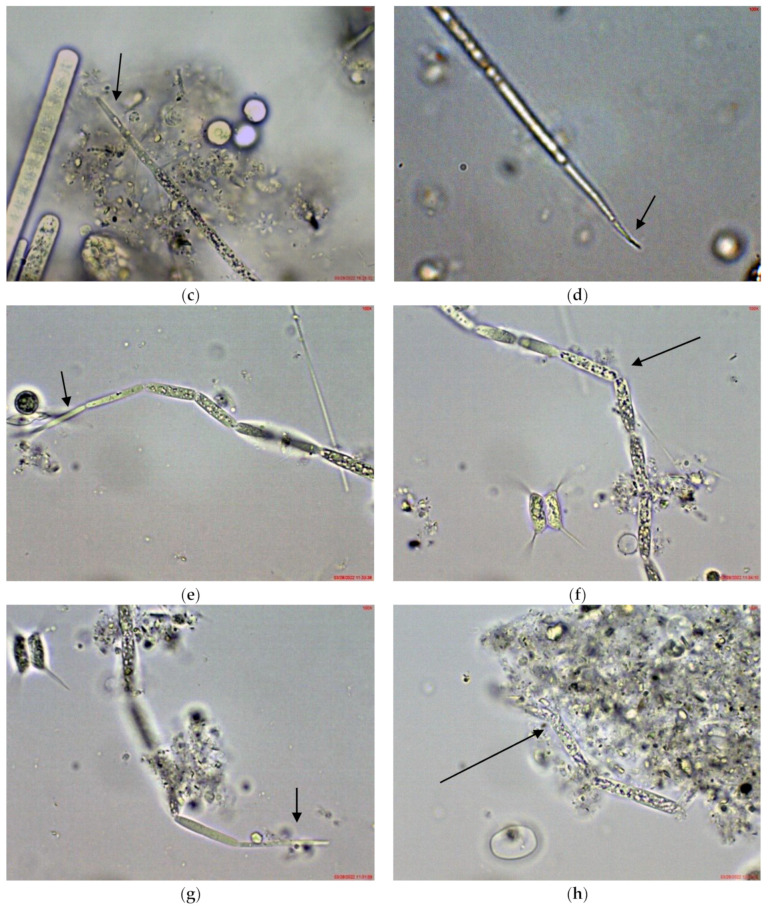
Examples of *Cuspidothrix* from the large coastal lake Vaya (western part, sites 1 and 2; 2018): (**a**) Part of a trichome with a well-developed intercalar heterocyte (arrow); (**b**–**d**) *Cuspidothrix issatschenkoi*—apical parts of three trichomes with typical vegetative cylindrical cells, not constricted at cell walls and differently developed apical cells (arrows); (**e**–**g**) young trichome with typical terminal cells (arrows on (**e**,**g**)) in a process of disintegration (arrow on (**f**)); (**h**) fragment of *Cuspidothrix* trichome reproducing through disintegration (arrow).

In the large, coastal Lake Vaya, specimens of *Cuspidothrix* were found in all studied sites, but in different quantities. In both sites 1 and 2, in samples collected from the western part of the lake, *C. issatschenkoi* was the most abundant, observed in different stages of development, often with heterocytes (Figure 1a) and non-matured akinetes. The morphological variation concerned mainly the length of apical cells, while the width of trichomes varied slightly from 2 to 3 (4) µm (Figure 1b–d). Some trichomes were young, non-heterocytous and sterile (i.e., without akinetes). Therefore, they could be only tentatively determined to species level, mainly on the basis of apical cells peculiar to each species. In addition, many trichomes were seen as vegetatively reproducing by disintegration in the typical for the genus “zig-zag” way (Figure 1e–g). Some fragments of disintegrating trichomes were also found in the samples (Figure 1h).

**Figure 2:**
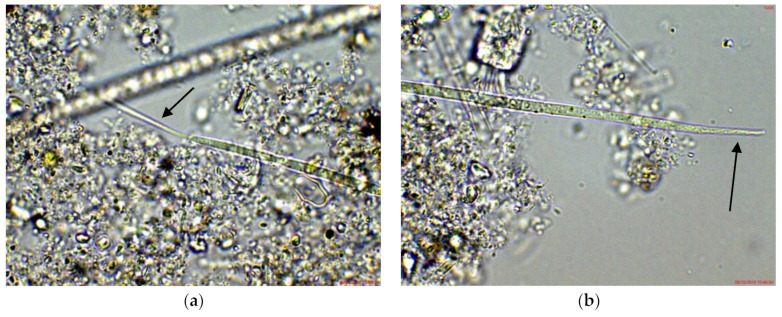
Example of *Cuspidothrix* from the large coastal lake—Vaya (eastern part, site 3; 2018): (**a**,**b**) both poles (arrows) of a young, sub-symmetric trichome of *C. elenkinii*.

In the eastern part of the lake (site 3), *Cuspidothrix* was also observed, but mainly in young stages without heterocytes or as disintegrating fragments. However, the morphological diversity in this site was higher: according to the apical cells, most of the specimens resembled *C. issatschenkoi*, several looked similar to *C. tropicalis,* and a few had the peculiar thin, “thread-like” cells of *C. elenkinii* in at least at one of the poles (Figure 2), thus being sub-symmetric in the terminology of Komárek [38].

**Figure 3:**
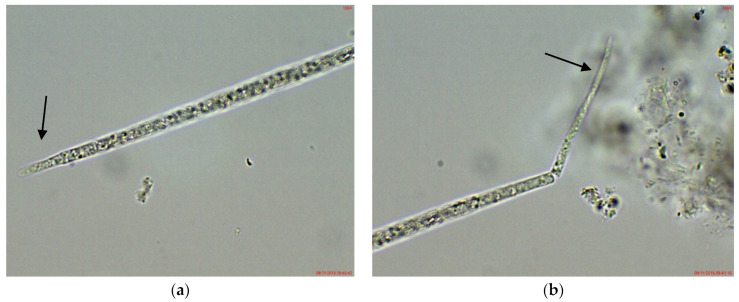
Example of *Cuspidothrix* from the small coastal reservoir Poroy (2018): (**a**,**b**) Both poles (arrows) of a young, sub-symmetric trichome of *C. tropicalis*.

In the small coastal reservoir Poroy, situated at a distance of about 30 km from Lake Vaya, *Cuspidothrix* was relatively rare, but was also found in different stages, mainly as young, undeveloped trichomes without heterocytes. Some of them were without aerotopes (groups of gas vesicles [38]) in the cells. Very young symmetric trichomes with pointed elongated ends looked quite similar to some young trichomes of the phylogenetically related genus *Raphidiopsis* [38]. Its species *R. raciborskii* (Wołoszyńska) Aguilera A., Berrendero Gómez E., Kaštovský J., Echenique R.O. et Salerno G.L. in the same period was abundant in Poroy [39]. According to the shape of the apical cells, some better developed individuals looked like *Cuspidothrix issatschenkoi* and some were close to *C. tropicalis* (Figure 3). However, in the absence of akinetes their identification was only tentative. All observed fragments of disintegrating trichomes strongly resembled the “zig-zag” ones recorded by us in Vaya, where *C. issatschenkoi* was abundant.

In the small inland reservoir Sinyata Reka, *Microcystis wesenbergii* (Komárek) Komárek ex Komárek dominated [40] and all other cyanoprokaryotes were quite rare. After obtaining the PCR results and additional processing of more than 10 slides from the concentrated sample, we managed to observe only several young trichomes without akinetes, similar to *Cuspidothrix*. Some of them had better-developed apical cells, which resembled *C. tropicalis,* and a few had very elongated apical cells, documented earlier for *C. issatschenkoi* (e.g., for its strain ORE27S2—Figure 9e in [41]) and recorded by us in some trichomes from Vaya (Figure 1c).

LM investigations of the summer phytoplankton samples collected in 2019 revealed a total of 128 species of Cyanoprokaryota. Out of them, 35 species occurred in the four reservoirs, from which strains containing ATX synthetase genes were recorded: 23 in Duvanli, 4 in Koprinka, 16 in Plachidol 2 and 6 in Sinyata Reka. Among all determined cyanoprokaryotes, 60 species belonged to the following 12 genera known as ATX-producers: *Anabaena*, *Aphanizomenon*, *Chrysosporum*, *Cuspidothrix*, *Dolichospermum*, *Microcystis, Oscillatoria*, *Phormidium*, *Planktothrix, Raphidiopsis, Sphaerospermopsis* and *Woronichinia*. As for 2018, below are discussed only results concerning findings of *Cuspidothrix*.

**Figure 4:**
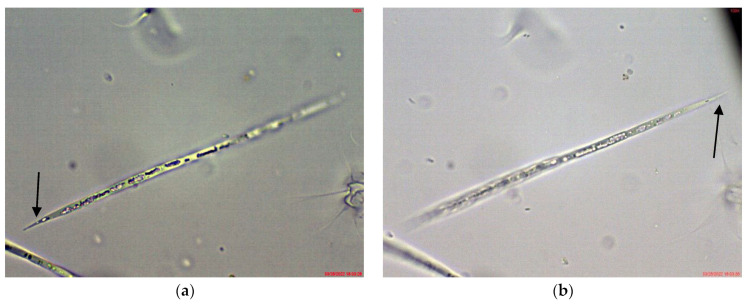
Example of *Cuspidothrix* from the small inland reservoir of Plachidol 2 (2019): (**a**,**b**) Both poles (arrows) of a young, symmetric, pointed trichome, comprised of cells containing aerotopes.

In the phytoplankton of the small inland reservoir Plachidol 2, dominated by *Anabaena* sp. ster., young symmetric trichomes with elongated, pointed apical cells and well developed aerotopes were rarely observed (Figure 4). They strongly resembled the young non-heterocytous trichomes of the genus *Raphidiopsis*, and of its species *R. mediterranea* Skuja, in particular. Due to lack of heterocytes and akinetes in all of them, their species identification by LM was impossible.

**Figure 5:**
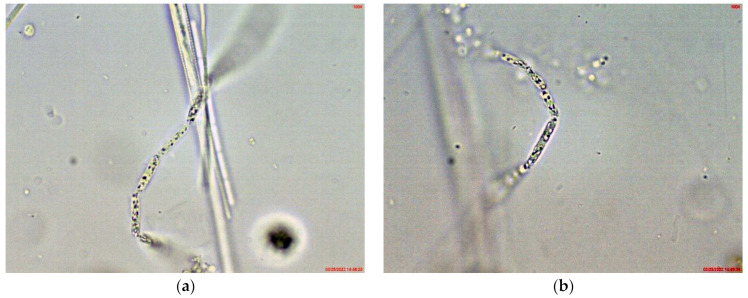
Examples of *Cuspidothrix* from the small inland reservoir of Duvanli (2019): (**a**,**b**) Fragments of disintegrating sterile trichomes.

*Cuspidothrix* was extremely rarely observed in the microscopic slides, processed from the small inland reservoir Duvanli, the phytoplankton of which at that time was polydominated by *Microcystis* sp. div., *Pseudanabaena limnetica* (Lemmermann) Komárek and *Romeria simplex* (Hindák) Hindák [42]. There, we found only several disintegrating trichomes, with typical “zig-zag” outfit of *Cuspidothrix* (Figure 5). Although in this stage reliable species identification was impossible, due to the identical morphology with the disintegrating trichomes found in the western part of Vaya (sites 1 and 2, 2018; Figure 1h) where *C. issatchenkoi* was abundant, we supposed that specimens from Duvanli may also belong to this species.

**Figure 6:**
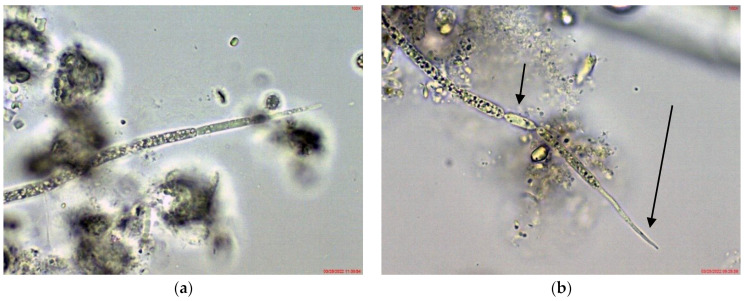
Example of *Cuspidothrix* from the small inland reservoir of Sinyata Reka and large inland reservoir Koprinka (2019): (**a**) apical part of the trichome of *Cuspidothrix tropicalis* from Sinyata Reka; (**b**) apical part of the trichome of *Cuspidothrix elenkinii* with well-developed apical cell (long arrow) and typical intercalar heterocyte (short arrow) from Koprinka.

*Cuspidothrix* was rarely found in the phytoplankton of the small inland reservoir Sinyata Reka, in which at that time *Sphaerospermopsis torques-reginae* (Komárek) Werner, Laughinghouse IV, Fiore et Sant’Anna dominated and *Microcystis wesenbergii* was relatively abundant [43]. The several observed specimens were without akinetes and rarely had heterocytes, but according to the typical apical cells resembled *C. tropicalis* (Figure 6a).

*Cuspidothrix* was a rare phytoplankter in the large inland reservoir Koprinka, where cryptophytes and diatoms dominated [42]. There, all observed trichomes of this genus had well-developed heterocytes and the peculiar “thread-like” apical cells typical of *C. elenkinii* (Figure 6b).

### 2.2. Results from Genetic-Molecular Studies

**Figure 7:**
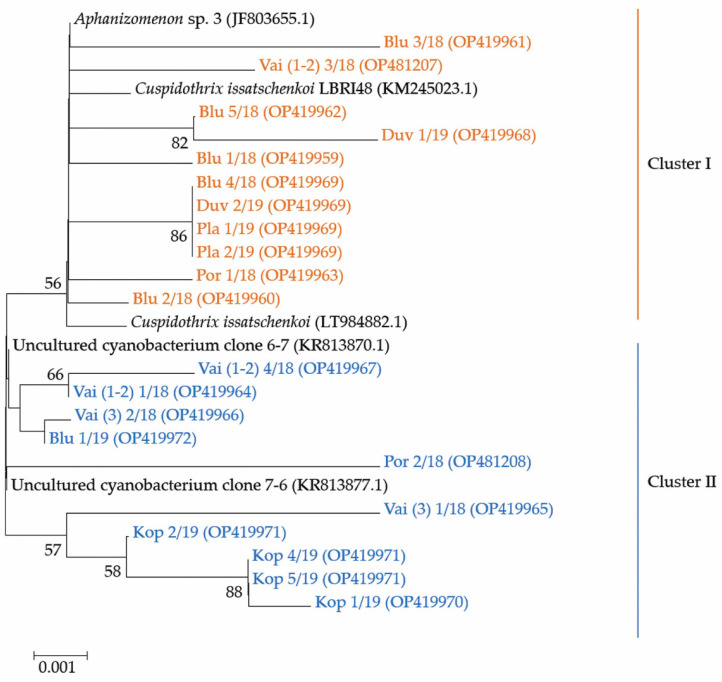
Neighbor-joining phylogenetic tree obtained after processing of phytoplankton samples from the summer phytoplankton of 6 Bulgarian waterbodies. The tree is constructed using 21 newly obtained nucleotides sequences (OP419959-OP419972, OP481207, OP481208) and closest sequences retrieved after BLAST [46] search in NCBI database [45] with indication of their original name, year of sampling and the relevant accession number in NCBI [45]. For the identical sequences (IS) only one accession number is provided: (i) The IS Duv 2/19, Blu4/18, Pla1/19, Pla 2/19 are with accession number OP419969; (ii) The IS Kop 4/19 and Kop 5/19, as well as the shorter sequence Kop 2/19 have accession number OP419971. Bootstrap values are shown at branch points (percentage of 1000 resamplings). Legend: Blu—Reservoir Sinyata Reka (=Blue River); Duv—Reservoir Duvanli; Kop—Reservoir Koprinka; Pla—Reservoir Plachidol 2; Por—Reservoir Poroy; Vai—Lake Vaya. For more details see the text of the paper.

Considering that ATX is constructed through the polyketide synthase pathway (PKS) by a cluster of at least eight *ana* genes [6,44], for the identification of different ATX producers in the environmental phytoplankton samples, we applied a general primer pair, designed for amplification of the specific *anaC* genes [5]. Resulting PCR signals were recorded in seven of the samples. They were most intensive in the samples from Lake Vaya and Sinyata Reka, collected in 2018, and from Koprinka, collected in 2019, whereas weak signals were registered in the samples from Poroy, collected in 2018, and from Duvanli, Plachidol 2 and Sinyata Reka, collected in 2019. By processing of these materials, 21 sequences representing 21 strains were identified. They were compared with genotypes available from the National Centre for Biotechnology Information (NCBI; Bethesda, MD USA) [45]. The phylogenetic analysis and the first constructed phylogenetic tree showed that all 21 strains sequenced during this study were clearly separated from the outgroup, composed of three “foreign” strains, observed by BLAST [46] analysis: *Oscillatoria* sp. PCC 10601 (JF803652; orig. Finland) and *Cylindrospermum stagnale* PCC7417 (CP003642.1; orig. USA), clearly distant from *Anabaena* sp. WA102 (CP011456.1; orig. USA: Anderson Lake). Because of the low homology (<88%), this outgroup is not shown on the part of the phylogenetic tree in Figure 7.

The phylogenetic analysis of the 21 sequences obtained by us revealed their grouping in two clusters (Figure 7). The first cluster contained eleven strains: nine were from the inland reservoirs Duvanli, Plachidol 2, and Sinyata Reka, and two strains were from the coastal reservoir Poroy and Lake Vaya. It is worthy to note that all strains, sequenced from Plachidol 2 and Duvanli, were only in this cluster, which comprised strains that showed high homology (>99%) with the available sequences of the identified species *Cuspidothrix issatschenkoi.* Seven of the eleven strains in this cluster were affiliated with *C. issatschenkoi* (LT984882.1) isolated from Norway, three were more similar to *C. issatschenkoi* LBRI48 (KM245023.1) from Lake Biwa (Japan), and only one strain (i.e., Blu 3/18, OP419961) could be tentatively related to the genus *Aphanizomenon* (JF803655.1) from Lake Sääskjärvi (Finland).

The second cluster contained 10 strains, sequenced from samples collected from the inland reservoirs Koprinka and Sinyata Reka, as well as from the coastal reservoir Poroy and Lake Vaya (Figure 7). These strains showed relatively high homology with two uncultured cyanobacterial strains, viz. clone 6-7 (KR813870.1) and clone 7-6 (KR813877.1) isolated from Dainchi Lake (China). Four of the strains were closer to clone 6-7 (KR813870.1) and six were affiliated with clone 7-6 (KR813877.1). Here, we would like to underline that all four sequences obtained from Koprinka were grouped only in this second cluster near clone 7-6 (KR813877.1).

## 3. Discussion

According to microscopic analysis by conventional LM of the phytoplankton samples from all 26 investigated Bulgarian lakes and reservoirs, 129 species of Cyanoprokaryota were determined. Out of them, 62 species (39 in 2018 and 60 in 2019), according to the literature, could be suspected as ATX producers. However, by application of genetic-molecular studies, we proved the presence of potential ATX producers only in seven samples, collected from three waterbodies in 2018 (Vaya, Poroy and Sinyata Reka) and from four waterbodies in 2019 (Duvanli, Koprinka, Plachidol 2, and Sinyata Reka). Only in one of them (Sinyata Reka) were ATX producers found in both sampling years, but with variance in toxigenic strains.

The differences in the occurrence of ATX producers in 2018 and 2019 could be explained by: (1) the dissimilarity in the environmental conditions during both campaigns. For example, the sampling in August 2019 was preceded by strong rains atypical for the country, which started in May and caused dilution of waters with decreased nutrient content and less pronounced algal blooms. By contrast, before the sampling campaign in June 2018, the weather was extremely hot and dry, which enhanced algal blooms (for details see [43]); (2) the shallow character of most Bulgarian waterbodies, in which frequent disturbances lead to a great algal diversity and rapid changes of dominants (e.g., [35,43]).

Regarding genetic diversity of the 21 toxigenic strains, identified in this study, and their coexistence, it is possible to state that highest diversity (five strains obtained in 2018) was detected in Lake Vaya, which is an important from conservational point of view waterfowl site on the bird migration route Via *Pontica* [35,47]. Similar diversity was recorded in the small inland strongly eutrophicated reserve Siniyata Reka, from which five strains were isolated in 2018 (but only one in 2019). According to the number of co-existing strains, the other waterbodies could be arranged in the following order: Koprinka (four strains), followed by Duvanli, Plachidol 2 and Poroy, with two strains isolated from each of them.

The results obtained by the applied PCR method revealed that algae containing the *anaC* gene belonged to the genera *Cuspidothrix* and *Aphanizomenon*. However, in 2005, based on genetic data, the genus *Cuspidothrix* was derived from the genus *Aphanizomenon* (with *C. issatschenkoi* formerly being known as *A. issatschenkoi* Kisselev—for details see [38,41]). Therefore, with a high level of probability it was supposed that the single sequence of unidentified species of *Aphanizomenon*, which appeared from BLAST [46] analysis nearby the sequences of *C. issatschenkoi*, in fact belonged to *Cuspidothrix*. Thus, we presumed that all potentially toxic cyanoprokaryote strains genetically identified in our study represented only the genus *Cuspidothrix,* three different species of which (i.e., *C. issatschenkoi*, *C. elenkinii* and *C. tropicalis*) were distinguished by LM in the relevant investigated samples.

Except in these samples, *Cuspidothrix* was discovered by LM also in the June 2018 phytoplankton of coastal reservoir Mandra (which together with Lake Vaya comprises a part of the large group of so-called Burgas lakes [35]), where it was rarely represented by single trichomes of *C. issatschenkoi,* and in the coastal lake Durankulak, where *C. tropicalis* occurred, forming <5% of the total biomass (for details see [39]). The lack of PCR signal for *anaC* genes in all samples from these two waterbodies, allows to suppose the atoxic character of their strains, or presence of other *ana* genes, which could not be covered by the used set of primers.

In general, the diazotrophic genus *Cuspidothrix,* which commonly bears specific heterocytes that contain nitrogenase (which is considered to be the responsible enzyme for the assimilation of gaseous atmospheric nitrogen (nitrogen fixation) [38]), was not commonly found in the studied waterbodies. Its rare occurrence and unequal distribution coincided with the relatively high content of TN detected during both sampling campaigns and is in accordance with the well-known decrease of the amount and diversity of heterocytous algae in rich in nutrient-eutrophicated waters (e.g., [48,49,50,51]). Since the facultative lack of heterocytes in environments rich in nitrogen has been documented [38] and toxigenic abilities of such non-heterocytous strains have been reported regarding ATX production [1,52,53,54], it is worthy to recall that in this study we found mainly non-heterocytous trichomes of *Cuspidothrix*. The only exception was the large reservoir Koprinka, in which TN was in low concentrations, and all observed specimens had well developed heterocytes (Figure 6b). Then, the finding of strains with toxigenic *anaC* gene in 16% of the investigated samples can be taken as an alert for potentially larger spread of ATXs in Bulgarian waterbodies, and especially in eutrophicated ones. There, the availability of nutrients, and the high nitrogen content in particular, may promote the production of ATXs, which contain nitrogen in their molecules [2,10,55,56].

Comparison of results achieved after application of LM and PCR methods, showed their general conformity. All genetic and morphological data pointed on the heterogeneity of *Cuspidothrix* in Bulgarian waterbodies, which once more demonstrates the great genetic diversity of cyanoprokaryote strains, highlighted in our previous works [39,40,43,57]. Considering biogeographical data on the origin of NCBI [45] strains affiliated with our material (Japan, China, Norway and Finland) the results from this study are in accordance with earlier conclusions on genetic similarity of European and Asian strains of the genus *Cuspidothrix* (e.g., [58]).

During the LM work, we found problems to distinguish reliably between young, non-heterocytous trichomes of *Cuspidothrix* and *Raphidiopsis* (preliminary *R. mediterranea*, but also *R. raciborskii*) especially in the reservoirs Poroy and Plachidol 2. Similar confusion between both genera was reported earlier and some subsequent genetic studies confirmed that strains, looking like *R. mediterranea* and its variety *grandis*, in particular, belonged to *Cuspidothrix* (e.g., [53,54,56,59,60]). On the other hand, we have to consider the long-lasting discussions on misidentifications and the validity of the genus *Raphidiopsis* (for details see [61,62]) and the fact that *R. mediterranea* is still a taxonomically accepted unit [63], which in the opinion of Moustaka-Gouni et al. [64] is clearly morphologically and genetically distinguishable from *C. issatschenkoi.* Therefore, we would like to highlight the need for further comparative LM and molecular-genetic studies of all species of *Raphidiopsis* and its phylogenetic relations with *Cuspidothrix.* At present, taking into account the obtained data on the spread of *anaC* genes in the sampled waterbodies, we accepted that at least a part of observed by LM trichomes in the reservoirs Poroy and Plachidol 2 belonged to *Cuspidothrix*. Moreover, on the constructed phylogenetic tree all four toxigenic strains from Poroy and Plachidol 2 were affiliated to *C. issatschenkoi* (Figure 7). It is the best studied and the most widely distributed species of the genus *Cuspidothrix* (e.g., [38,63]).

Beside these four strains, seven other strains sequenced from Duvanli, Sinyata Reka and Vaya, also fitted to *C. issatschenkoi.* This species was recorded by LM in the same waterbodies and its morphological variation, observed by LM, generally corresponded to the detected genetic diversity of strains. Here we would like to note particularly the case of disintegrating trichomes, found in Duvanli, Poroy and Vaya, which on the basis of their morphological similarity in LM, were presumed as belonging to *C. issatschenkoi.* In accordance with this conclusion, the strains sequenced from these three waterbodies, grouped in the same subcluster of the phylogenetic tree, showing high similarity to *C. issatschenkoi* (Figure 7). We would like to mention also that frequent disintegration of trichomes was recorded during the LM study of this species from Nakdong River of South Korea [60].

Ten strains obtained during this study from Koprinka, Poroy, Sinyata Reka and Vaya, grouped tightly with uncultured cyanobacterium clone 6-7 (KR813870.1) and uncultured cyanobacterium clone 7-6 (KR813877.1). In the same waterbodies, by LM we distinguished *C. elenkinii* and *C. tropicalis*, for which, according to our best knowledge, no sequences are available from NCBI [45].

At present, taking into account all abovementioned considerations from comparison of our LM data and clear groups of *Cuspidothrix* strains identified by PCR method, and following the recent taxonomy of *Cuspidothrix* with five accepted species names [63], we concluded that at least three species, i.e., *C. issatschenkoi*, *C*. *elenkinii* and *C*. *tropicalis*, were potential ATX producers in the studied Bulgarian lakes and reservoirs. In respect to their distribution in Bulgaria, we would like to note that:

- *C. issatschenkoi* has already been recorded by LM from “standing and running waters in Sofia region,” in the lakes Vaya, Durankulak and Sreburna, as well as in the reservoirs Burzina, Mandra and Poroy [39,65,66,67,68,69]);
- *C. elenkinii* has been reported from the reservoirs Aheloy, Antimovo, Boyka, Devets, Dubnika, Koprinka, Ovchi Kladenets, Poroy, Rabisha, Rasovo, Telish, Vulchovets, and Yastrebino [70], but was not found in a subsequent study of the phytoplankton dynamics of the reservoir Koprinka [71];
- Our first finding of *C. tropicalis* in Bulgaria in June 2018 (in Vaya, Durankulak and Poroy) was already documented by its inclusion in the list of potential producers of another important cyanotoxin—cylindrospermopsin [39]. However, there we had not published more detailed LM and the supporting genetic data, which we provide in this paper, extending information on its occurrence by data from samplings conducted in 2019. In addition, here, based on our long expertise in phytoplankton studies in Bulgarian waterbodies, we would like to underline the presumed alien character of this tropical species. This statement is strongly supported by the fact that it was observed mainly in coastal waterbodies along the Black Sea, which provide resting and nesting sites on the important bird migration route Via *Pontica,* used by waterfowl and other species flying back from tropical Africa [47].

In conclusion, the present work provides the first genetically proved data on the occurrence and distribution of the potentially toxic strains of cyanoprokaryote genus *Cuspidothrix* in Bulgaria, demonstrating presence of ATX synthetase gene *anaC* in 21 strains, spread in five reservoirs and one natural lake of the country. Considering that all cyanotoxins are of rising global concern [72,73], pose serious threat to the vulnerable Bulgarian waterbodies [35,74] influencing both environmental and human health strongly related with national security [75], further exploration is motivated to clarify the current spread and toxigenicity of cyanoprokaryotes in the whole country. Therefore, future enlargement of the monitoring of the waterbodies in the country combined with chemical detection of ATX and ATXs is strongly needed. The results may provide further insights to the biogeography of globally important ATX producers, but it has to be stressed that such comprehensive information is crucially important for achieving reliable risk assessment of Bulgarian waterbodies and for taking of adequate measures in a timely manner.

## 4. Materials and Methods

### 4.1. Studied Sites and Field Sampling

**Figure 8:**
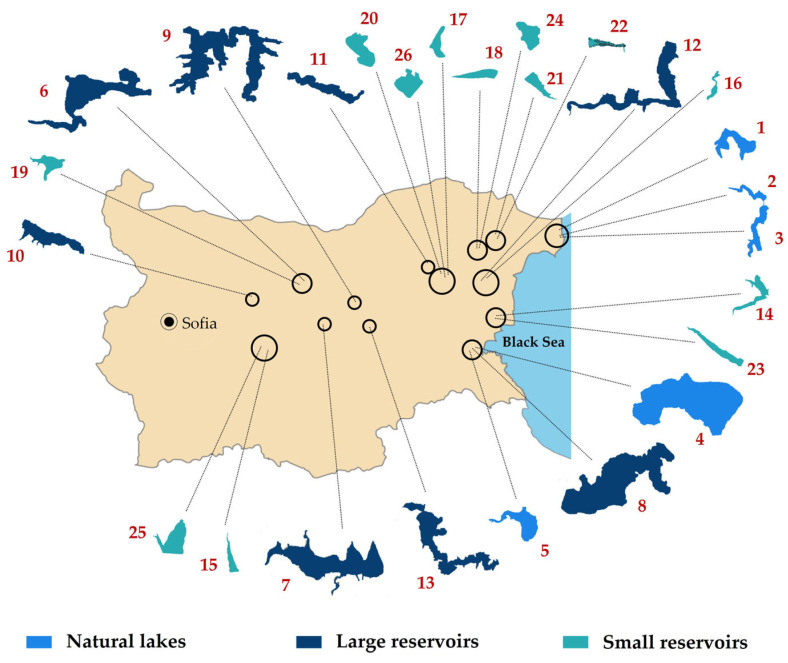
Map of Bulgaria with indication of the locations and shapes of all sampled 26 waterbodies. Numbers follow those in Table 1. For more details see the text of the paper.

The study was based on 44 processed surface phytoplankton samples from 26 selected waterbodies in Central and Eastern Bulgaria. Sampling campaigns were conducted in June 2018 (17 sites of nine waterbodies) and in August 2019 (27 sites of 25 waterbodies)—Table 1, Figure 8. Detailed hydro-morphological data and descriptions with notes on their use and conservational status are available from the Inventory of Bulgarian wetlands [35] and, therefore, the identification number from inventory’s database (IBWXXXX) is provided in Table 1.

Due to the specific aim to search for algal blooms, in both years sampling was preceded by sending of a drone with a camera (DJI Mavic Pro, Model M1P GL200A; SZ DJI Technology, Shenzhen, Guangdong, China, in 2018, and DJI Mavic 2 Enterprise Dual; Shenzhen, Guangdong, China, in 2019). The observations of the aquatories of the visited wetlands aimed to discover and document the visible accumulations of cyanoprokaryotes (blooming areas or spots) distinguished by changes in the water color [34]. Subsequently, the chosen sites were reached by inflatable boats. In cases when algal accumulations were not visible, samples were collected at sites, visited in our previous studies. Geographical and physical parameters, such as altitude, coordinates, water temperature, water pH, water hardness (TDS), oxygen content (DO), and conductivity, were measured in situ with Aquameter AM-200 and Aquaprobe AP-2000 from Aquaread water monitoring instruments, 2012 Aquaread Ltd. (Broadstairs, UK), whereas the transparency was estimated according to Secchi disk data (Table 1). The main nutrients, total nitrogen (TN) and total phosphorus (TP) were measured ex situ using an Aqualytic AL410 photometer from Aqualytic^®^ (Dortmund, Germany) at the first possibility after collection (Table 1).

At each site, phytoplankton samples were collected for genetic and molecular studies (viz. PCR) and for taxonomic identification by LM. All samples were taken from the surface layer (0–20 cm) in a volume of 0.5 or 1 L, depending on the water color intensity, with increasing volume at brighter color of the water. The samples for algal determination and enumeration were fixed with 2–4% formalin at the site and transported to the lab, where they were concentrated by sedimentation, lasting minimum 48 h and further processed by LM. The samples for PCR were filtered ex situ at the first possibility after collection, and the obtained filters, kept in sterile plastic tubes, were transported to the lab in a dry ice.

### 4.2. Microscopic (LM) Processing of the Phytoplankton Samples and Species Identification

The LM work was carried in the lab using Motic BA and Motic B1 microscopes (Wetzlar, Germany), with a Moticam 2000 and Moticam 2.0 mp camera, both supplied by Motic Images 2 and 3 Plus software program, respectively.

The identification of algae was done on non-permanent slides under magnification 100× with application of immersion oil and was based on standard European taxonomic literature consulted with recent data in AlgaeBase [63] and relevant papers ([41,61,76], etc.). Regarding Cyanoprokaryota, the three volumes of the Freshwater Middle European Flora were used as main determination source [38,77,78]. It has to be noted that LM work was conducted twice: first, in 2018–2019 after each of the sampling campaigns, when from each site at least two slides were processed, and then, in 2021–2022, it was repeated on more slides with special attention to the sites, in which toxin producers had been detected genetically.

For morphological identification of *Cuspidothrix* species in particular, the following diagnostic features were followed: (1) shape and size of vegetative cells; (2) presence/absence of constrictions at cell walls; (3) presence or absence of mucilage sheath; (4) shape and size of apical cells; (5) shape, size and position of heterocytes; (6) shape, size and position of the specific resting and reproductive cells—akinetes [38]. In the text, the specific term “trichome” was used to name the organization of cells in a thread-like unit without a mucilage sheath, since “filament” in cyanoprokaryote studies is commonly applied in cases when trichome is surrounded by such a sheath (e.g., [38]).

The potential ATX and ATXs producers among the planktic cyanoprokaryotes were evaluated according to the data provided by [1,3,79]. The term “alien” was used for non-native, allochthonous species [80,81,82,83,84].

### 4.3. Molecular and Genetic Studies

The frequent spread of eight ATX synthetase genes (*ana* genes) in both sediment and water samples was shown by Legrand et al. [85], who were the first to assess the molecular tools for detection of ATXs in waters. Sequencing of the *ana* gene clusters of different strains of *Cuspidothrix issatschenkoi* demonstrated that their arrangement patterns were identical, but differed from those of *Oscillatoria* and *Anabaena,* except for the conserved section with *anaB-anaG* genes [57]. Therefore, *anaC* was amplified using a general primer set, originally designed for multi-generic detection of different ATX- and ATXs-producing genera [5]. The primer sequences used were F-ATGGTCAGAGGTTTTACAAG and R-CGACTCTTAATCATGCGATC [5].

Standard reactions (25 μL) were performed with 12.5 μL MyTaqHS Mix (Bioline, London, UK), which included 10 pmol (1 μL) primers straight and inverted. The reaction mixtures were incubated in a QB-96 Thermal Cycler using the following program: denaturation at 95 °C for 3 min, 35 cycles of denaturation (10 s at 95 °C), annealing at 52 °C for 30 s, extension at 72 °C for 30 s, and a final extension at 72 °C for 5 min. The obtained *anaC* PCR products were purified and cloned using GeneJET™ Thermo Scientific and Clone JET PCR purification kits for cloning (Thermo Fisher Scientific, Waltham, MA, USA). Then the recombinant clones were sent to Macrogen Europe (Amsterdam, The Netherlands), where they were Sanger sequenced with the same pJET primers. Depending on the obtained PCR product intensity, different number of clones per sample were sent to Macrogen Europe (Amsterdam, The Netherlands) for sequencing: four or five *anaC* clones per sample were send in cases of intensive PCR signal, while at a weak PCR amplification, only one or two clones were used for sequencing. Obtained data were manually edited and initially analyzed using Vector NTI 11.5 (Thermo Fisher Scientific, Waltham, MA, USA) software package. Afterwards, a phylogenetic tree was constructed using Mega 6.0. program [86] and the Neighbor-Joining method with 1000 bootstrap values. The obtained *anaC* sequences were deposited in the NCBI GenBank database [45] under the accession numbers OP419959—OP419972, OP481207, and OP481208.

## Figures and Tables

**Table 1:** Sampling sites in Bulgarian waterbodies and their environmental parameters during both sampling campaigns of June 2018 and August 2019. First are enlisted the natural lakes, followed by the large reservoirs, and small reservoirs, all organized in alphabetical order. Legend: WBN—name of the water body; IBW—number in the Inventory of Bulgarian Wetlands [35]; E—Eastern part of the waterbody, W—western part of the waterbody (the nearest site from the former year is additionally labelled with one of these letters); Res—reservoir; L—lake; Site—number or name of given site, only in case of more than one sampled sites; Alt—altitude above the sea level (m); WT—water temperature (°C), SD—Secchi disk Secchi disk transparency (m); TTB—total transparent to bottom; CN—conductivity (S m^−1^); TDS—total dissolved solids (µg L^−1^); DO—oxygen concentration (mg L^−1^); TP—total phosphorus (mg L^−1^); TN—total nitrogen (mg L^−1^).

WBN and IBW	Site	Year	Alt	Latitude	Longitude	WT	pH	SD	CN	TD	DO	TP	TN
Natural Lakes													
1. L. Durankulak (IBW0216)	1	2018	6	43°40.3240′	28°32.0470′	24.03	8.5	1	1111	722	7.35	21	2.8
	2	2018	6	43°40.3340′	28°32.0220′	24.7	8.2	1	1094	711	7.79	20	4.0
	3/W	2018	4	43°40.5300′	28°32.9930′	24.6	8.5	1	1075	698	6.19	24	3.9
	W	2019	2	43°40.0006′	29°32.6166′	26.5	8.9	0.6	974	631	7.86	0.3	0.7
	4/E	2018	3	43°40.6950′	28°32.6000′	26.5	8.5	1	1087	706	9.6	20	3.2
	E	2019	4	43°40.5355′	28°33.0806′	26.7	8.9	0.6	1048	680	6.04	0.3	0.6
2. L. Ezerets (IBW0233)		2018	−2	43°35.2770′	28°33.2290′	26.4	8.4	TTB	1084	0	9.94	0.5	5.3
		2019	6	43°35.2681′	28°33.2096′	25.9	8.6	1.5	1669	1739	8.58	0.1	0.1
3. L. Shabla (IBW0219)		2018	−2	43°33.8180′	28°34.1860′	27.1	8.5	TTB	1087	706	9.97	0.1	5.1
		2019	<1	43°33.8212′	28°34.8204′	25.9	8.7	TTB	1842	1196	9.64	0.1	1.0
4. L. Vaya (IBW0191)	1	2018	−2	42°30.5940′	27°22.075′	26.9	9.7	0.25	2588	1682	12.51	13	5.4
	2/W	2018	0	42°28.4540′	27°25.482	28.28	8.9	0.25	1183	768	11.94	11	3.7
	W	2019	−2	42°30.5940′	27°22.075′	27.9	9.2	0.15	490	17	7.69	0.5	0.3
	3	2018	6	42°29.1850′	27°26.531	23.7	9.5	0.25	1024	665	7.01	12	4.6
5. L. Uzungeren (IBW0710)		2018	7	42°26.1782′	27°27.1998′	25.9	8.1	0.40	1458	9351	7.83	5.0	2.8
		2019	−3	42°26.1551′	27°27.2235′	27.6	8.5	0.45	1748	1132	9.7	0.4	0.3
Large reservoirs													
6. Res. Al. Stamboliyski (IBW2056)		2019	190	43°07.0000′	25°07.3936′	29.4	8.9	2.00	670	4.33	9.82	1.4	3.5
7. Res. Koprinka (IBW2062)		2019	450	42°37.0172′	25°19.4795′	27.2	8.2	2.5	250	163	7.21	0.1	0.2
8. Res. Mandra (IBW1720)	1	2018	12	42°24.0643′	27°26.1120′	25.9	8.3	0.4	649	421	6.81	3.0	3.0
	2/W	2018	13	42°24.0670′	27°19.1310′	26.2	8.2	0.2	663	461	5.89	6.0	4.0
	W	2019	7	42°24.0295′	27°19.1194′	25.88	7.9	0.45	676	436	7.93	0.7	0.5
	3/E	2018	9	42 26.1420′	27°26.5860′	24.9	8.5	0.3	639	415	7.91	4.0	3.3
	E	2019	8	42°25.9303′	27°26.7652′	27.2	8.5	0.45	578	375	7.87	1.5	1.8
9. Res. Shilkovtsi (IBW2105)		2019	410	42°55.2320′	25°47.6743′	27.2	8.9	0.5	746	479	7.48	0.03	0.1
10. Res. Sopot (IBW1437)		2019	376	40°00.7017′	24°52.6045′	29.0	8.3	2.0	779	490	3.44	0.1	0.1
11. Res. Suedinenie (IBW2642)		2019	133	43°20.0734′	26°33.6368′	28.1	7.6	0.5	739	481	6.77	0.1	0.3
12. Res. Tsonevo (IBW3022)		2019	75	43°01.8055′	27°24.3965′	24.8	8.8	4.2	355	231	8.2	0.1	0.1
13. Res. Zhrebchevo (IBW2545)		2019	253	42°36.6024′	25°51.2345′	27.6	7.7	0.7	358	233	8.01	0.1	0.2
Small reservoirs													
14. Res. Aheloy (IBW3032)		2018	144	42°42.8230′	27°30.9740′	25.4	8.5	1.10	614	399	8.92	1	4.1
15. Res. Duvanli (IBW1483)		2019	250	42°23.1851′	24°43.1000′	26.3	8.8	0.4	4050	291	7.09	0.1	0.3
16. Res. Eleshnitsa (IBW3023)		2019	44	43°00.3344′	27°28. 0744′	26.7	8.4	2.00	532	347	6.78	0.1	0.3
17. Res. Fisek (IBW2674)		2019	182	43°18.8453′	26°44. 3765′	27.2	8.7	0.5	690	3.97	7.52	0.2	0.1
18. Rez. Izvornik 2 (IBW3082)		2019	255	43°27.3838′	27°21.111′	24.5	9.4	0.15	389	253	13.26	9.0	4.8
19. Res. Krapets (IBW2000)		2019	410	43°04.0316′	24°52.3835′	28.7	8.3	5.0	870	564	7.74	0.1	1.0
20. Res. Kriva Reka (IBW3071)		2019	133	43°22.6573′	27°10.9807′	23.7	8.4	0.3	662	428	6.24	1.0	9.0
21. Res. Malka Smolnitsa (IBW3107)		2019	211	43°36.2606′	27°44.5367′	25.2	9.1	0.3	755	490	7.05	0.6	0.6
22. Res. Plachidol 2 (IBW5073)		2019	220	43°33.3504′	27°52.6338′	24.6	9.0	0.5	1225	793	9.13	0.2	0.4
23. Res. Poroy (IBW3038)		2018	41	42°43.0190′	27°37.3160′	25.10	8.3	1.2	762	495	9.45	1.0	2.8
		2019	43	42°43.3403′	27°37.5255′	27.5	8.1	0.4	644	416	7.6	0.1	0.3
24. Res. Preselka (IBW3078)		2019	281	43°25.3767′	27°16.6214′	24.1	9.0	0.5	138	282	10.05	0.6	2.8
25. Res. Sinyata Reka (IBW1890)		2018	317	42°28.1480′	24°42.2170	27.4	9.7	0.5	470	305	9.36	25	4.8
		2018	317	42°28.1473′	24°42.2175	26.7	9.4	0.6	468	306	9.21	27	4.3
		2019	317	42°28.1518′	24°42.0159′	28.2	10.4	0.4	490	317	14.76	1.0	0.2
26. Res. Shumensko Ezero (IBW2754)		2019	152	43°14.8140′	26°57.5675′	25.2	8.5	1.0	471	445	6.32	0.2	0.5

## Data Availability

Data supporting reported results can be found in the NCBI GenBank database (https://www.ncbi.nlm.nih.gov, accessed on 26 September 2022) under the accession numbers OP419959-OP419972, OP481207, and OP481208.
